# “Just right” combinations of adjuvants with nanoscale carriers activate aged dendritic cells without overt inflammation

**DOI:** 10.1186/s12979-023-00332-0

**Published:** 2023-03-09

**Authors:** Ananya Ananya, Kaitlyn G. Holden, Zhiling Gu, Dan Nettleton, Surya K. Mallapragada, Michael J. Wannemuehler, Marian L. Kohut, Balaji Narasimhan

**Affiliations:** 1 Nanovaccine Institute, Iowa State University, Ames, IA 50011 USA; 2 Department of Chemical and Biological Engineering, Iowa State University, Ames, IA 50011 USA; 3 Department of Statistics, Iowa State University, Ames, IA 50011 USA; 4 Department of Kinesiology, Iowa State University, Ames, IA 50011 USA

**Keywords:** Immunosenescence, Inflammation, Dendritic cells, Vaccines, Pattern recognition receptors, Nanoparticles, Micelles, Cytokines, Principal component analysis

## Abstract

**Background:**

The loss in age-related immunological markers, known as immunosenescence, is caused by a combination of factors, one of which is inflammaging. Inflammaging is associated with the continuous basal generation of proinflammatory cytokines. Studies have demonstrated that inflammaging reduces the effectiveness of vaccines. Strategies aimed at modifying baseline inflammation are being developed to improve vaccination responses in older adults. Dendritic cells have attracted attention as an age-specific target because of their significance in immunization as antigen presenting cells that stimulate T lymphocytes.

**Results:**

In this study, bone marrow derived dendritic cells (BMDCs) were generated from aged mice and used to investigate the effects of combinations of adjuvants, including Toll-like receptor, NOD2, and STING agonists with polyanhydride nanoparticles and pentablock copolymer micelles under in vitro conditions. Cellular stimulation was characterized via expression of costimulatory molecules, T cell-activating cytokines, proinflammatory cytokines, and chemokines. Our results indicate that multiple TLR agonists substantially increase costimulatory molecule expression and cytokines associated with T cell activation and inflammation in culture. In contrast, NOD2 and STING agonists had only a moderate effect on BMDC activation, while nanoparticles and micelles had no effect by themselves. However, when nanoparticles and micelles were combined with a TLR9 agonist, a reduction in the production of proinflammatory cytokines was observed while maintaining increased production of T cell activating cytokines and enhancing cell surface marker expression. Additionally, combining nanoparticles and micelles with a STING agonist resulted in a synergistic impact on the upregulation of costimulatory molecules and an increase in cytokine secretion from BMDCs linked with T cell activation without excessive secretion of proinflammatory cytokines.

**Conclusions:**

These studies provide new insights into rational adjuvant selection for vaccines for older adults. Combining appropriate adjuvants with nanoparticles and micelles may lead to balanced immune activation characterized by low inflammation, setting the stage for designing next generation vaccines that can induce mucosal immunity in older adults.

**Supplementary Information:**

The online version contains supplementary material available at 10.1186/s12979-023-00332-0.

## Background

Immunosenescence is a process of immune system dysfunction associated with aging. It is a complex phenomenon that can have major impacts on both innate and adaptive immunity, influencing chronic illnesses [1–3]. Immunosenescence is associated with a diminishing capacity to mount an effective immune response and the senescence of immune cells [4]. This cellular senescence is a hallmark of aging, wherein cells cease to divide and undergo an irreversible cessation of replication [4, 5]. The senescence-associated secretory phenotype (SASP) of senescent cells is characterized by the release of proinflammatory cytokines and matrix-degrading molecules. Disproportionate production of stimulating mediators like cytokines, chemokines, and acute phase reactants, as seen with SASP, often leads to a persistent, systemic, low-grade inflammatory condition known as “inflammaging” [6–8]. Inflammaging is multifactorial, driven to some extent by damaged organelles, reduction of autophagy, increased danger/damage-associated molecular pattern (DAMPS) [8] that contribute to chronic stimulation of the innate immune system [7] which is followed by a rise in senescent cell accumulation, and the production of proinflammatory cytokines [9–12]. It has been demonstrated that greater levels of inflammatory cytokines secreted by immune cells is associated with poor vaccination response [13, 14]. Some studies have also shown that preexisting inflammation could lead to poor vaccine efficacy in multiple vulnerable populations (e.g., older adults, people with autoimmune disorders) that may most critically need vaccination [14–17].

Dendritic cells (DCs) are a heterogeneous collection of antigen-presenting cells (APC) that play a crucial role in vaccination because they prime T cell immune responses against antigens via a peptide-MHC interaction (Signal 1) [18]. When triggered by microbial-associated molecular patterns (MAMPs) via the interaction with pattern recognition receptors (PRRs) such as Toll-like receptors (TLRs) [19, 20], intracytoplasmic NOD-like receptors (NLRs) [21, 22] and C-type lectin receptors (CLRs) [23, 24], DCs upregulate costimulatory molecule expression (Signal 2) and produce cytokines (Signal 3) that contribute to T cell activation [25]. The extent of DC costimulatory molecule expression and cytokine profile shape T cell activation and memory [26]. However, the function of DCs in linking innate and adaptive immune response in older adults is not well understood [27]. It has been shown that with aging, DCs are less effective in processing and presenting antigens to T cells [28–31] and their cytokine output is not optimal for stimulating potent adaptive immune responses [32, 33]. Therefore, to optimize vaccine design for older adults, it would be useful to identify strategies aimed at enhancing the functionality of DCs [32]. Vaccines that are directed towards DC stimulation provide a wealth of options for modulating humoral immune responses, including fine-tuning T cell polarization and managing antigen accessibility for B cells [34]. Adjuvants (e.g., biomaterials, synthetic materials, viral vectors) are immunostimulants that are added to vaccines to increase and improve the amplitude and duration of the immunological response. Adjuvants initiate effector functions of APCs by inducing an inflammatory profile [35, 36]. Immunosenescence and the age-related rise in the proinflammatory state may increase susceptibility to infection and reduce vaccine responsiveness. Effects of inflammaging are present within tissues including the lung, which must be taken into account in the development of vaccines for mucosal sites [37]. Therefore, vaccines targeted at enhancing the aged immune system must simultaneously seek to mitigate the inflammatory state and optimize adaptive immunity [38]. It is possible that suppressing or modifying inflammation, as opposed to eliminating it, might provide a unique opportunity to induce potent immune responses in aged populations. To improve vaccine-induced immunity, it would be beneficial to create new “combination vaccines” that can either broadly or selectively balance and/or channel certain basal inflammation pathways [35].

Effective stimulants are needed to counteract the low-grade inflammatory state that may impede vaccination responses, as well as to improve innate and adaptive immune responses to vaccines and to provide long term protection against infection in aged individuals [39–41]. To improve immunogenicity in older adults, polymeric adjuvants may be modified to include immunomodulatory drugs [42], and polymer chemistry can be tailored to regulate protein release kinetics [43]. In this context and to combat age-related deficits in vaccination response, we have created two effective and safe vaccine delivery systems based on biodegradable polyanhydride nanoparticles composed of 1,8-bis(*p*-carboxyphenoxy)-3,6-dioxaoctane (CPTEG) and 1,6-bis (*p*-carboxyphenoxy) hexane (CPH) [44–46] and self-assembling nanoscale amphiphilic poly(diethyl aminoethyl methacrylate) (PDEAEM)-Pluronic pentablock copolymer (PBC) micelles [46]. Both platforms induce ‘optimal’ inflammation, and from the perspective of aging, are complementary based on the observations that nanoparticles enhance T cell immunity [47] and micelles boost B cell responsiveness [48]. We have shown that PBC micelles and nanoparticles, unlike TLR ligands, induce much less secretion of inflammatory cytokines by DCs [49]. Nanovaccines can be designed by judiciously combining adjuvants and antigens with nanoparticles and micelles. In comparison to traditional vaccine adjuvants, such as alum, nanovaccines offer significant benefits, including improved thermal stability, decreased reactogenicity, and increased shelf-life stability of the payload [50]. The polyanhydride-based Gliadel® wafer has been approved for use in humans [51], and we have shown that polyanhydride nanoparticles have excellent tissue compatibility in mice [52]. Additionally, there is no indication of toxicity, inflammation, or necrosis at the injection site when using PBC micelles in mice [53]. At body temperature (37 °C), high concentrations of polymer micelles in aqueous solution produce physical gels that entrap proteins and allow for long-term antigen delivery [54–57]. Amphiphilic regions improve cellular uptake [58, 59], while the Pluronic block encourages endocytosis [60]. PDEAEM has also been shown to enhance adjuvanticity [61, 62].

In this study, we tested several PRR-dependent adjuvants together with our two polymeric “adjuvant nano-carriers” (i.e., 20:80 CPTEG:CPH nanoparticles (NPs) and PBC micelles (Mi)) as a means of activating aged bone marrow derived DCs (BMDCs). The rationale for the choice of these adjuvants and the types of immune responses induced by them are outlined in Table 1. Because each PRR adjuvant can impact different mechanisms, compensating for the impaired innate and adaptive immunity of older adults, their targeted usage, either alone or in combination, will likely be vital in future vaccine development [27]. Our objective was to identify adjuvant/biomaterial combination(s) that appropriately stimulate aged BMDCs by elevating costimulatory molecule expression (CD40, CD80, CD86) and inducing cytokines (IL-12p70, IFNα, IL-6, IFNβ, IFNγ) associated with T cell activation (CD4^+^ T_fh_ and CD8^+^ memory) while promoting “optimal” inflammation without excessive production of IL-1β, IL-6,TNFα, and IL-12p40.

**Table 1:** Experimental stimulants, their immunological function, and dosage used

	Immune stimulant	Agonist	Immune response	Dosage
1	Class B CpG oligonucleotide	TLR9 agonist	Promotes activation and maturation of plasmacytoid DCs (pDCs) and stimulate small amount of INFα and IFNβ [63] Strongly activates B cells [64, 65] Induces secretion of IL-6, IL-10, IL-12 [66]	5 μg/mL
2	Bis-(3′-5′)-cyclic dimeric guanosine monophosphate (c-di-GMP) (CDN)	STING Agonist	Increases production of IL-12, IFN-γ, IL-8, MCP-1, and RANTES Enhances T cell stimulatory activity [67]	1 μg/mL
3	Flagellin from *B. subtilis* (FLA-BS)	TLR5 [68], NLRs, NLRC4 and NAIP5 [69]	Extracellular FLA-BS activation through TLR5 triggers MyD88- dependent-B activation, cytokine, and NO generation [70]. Detection of intracellular flagellin by NLRC4 and NAIP5 results in the formation of an inflammasome, which activates caspase-1 of IL-1β and IL-18 [71, 72].	100 ng/mL
4	Lipopolysaccharides (LPSs)	TLR2 and TLR4	Powerful T cell adjuvant that promotes clonal expansion of B cells, resistance to growth factor deprivation, and Th cell differentiation of activated T cells. Induces production of interlukin-1β (IL-1β), IL-6, IL-10, IL-12, and TNFα [73]	1 μg/mL
5	Monophosphoryl-Lipid A (MPLA)	TLR 4	Induces a strong Th1 response Promotes IFN-γ production by Ag- specific CD4 T cells [74] Enhance production of antigen-specific CD8 cytotoxic T lymphocytes [75] Induces production of IL-1β, IL-10, IL-6, IL-8, and TNF-α [73]	1 μg/mL
6	Muramyl dipeptide (MDP)	NOD2	MDP increases the production of IL-10 when coupled with TLR2 agonists [76] MDP’s potential stimulation of NLRP3 and NLRP1 produces IL-1β [77] Combining MDP with LTA (a TLR2 agonist) stimulates human monocyte derived DCs and increases TNF-α and IL-12 secretion [78]	1 μg/mL
7	R848	TLR7 and TLR 8	R848 strongly induces cytokines such as TNF-α, IFNα, IL-12, and IFN-γ Helps in the induction of Th1 and Th2 cell types [79].	100 ng/mL
8	Polyanhydride NP	–	NP are readily phagocytosed by DCs, allow sustained release of antigen, and show a safe toxicological profile [80, 81] Induce T cell responses Has been shown to cause low inflammation [49]	100 μg/mL
9	PBC Micelles	–	Micelles offer pH-responsive micellization and gelation to aid the antigen endosomal escape in a sustainable manner Induce B cell responses Has been shown to cause low inflammation [82]	12.5 mg/mL

## Results

### Upregulation of costimulatory molecule expression by adjuvants

**Fig. 1:**
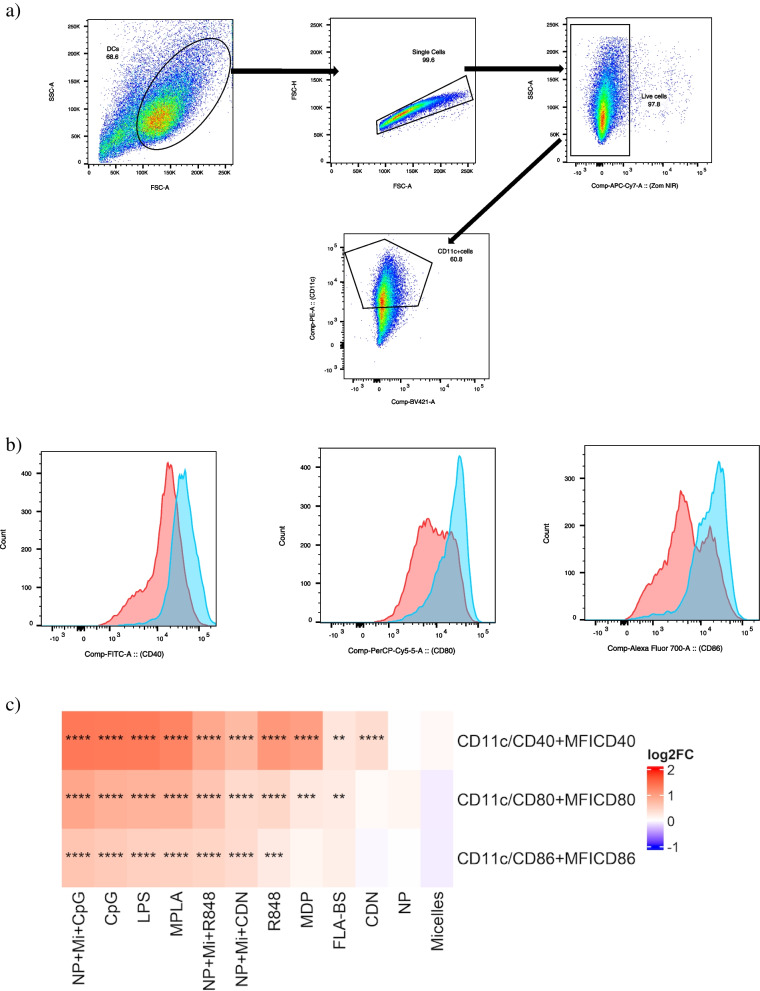
**a** Gating strategy used in flow cytometric analyses to gate the cells of interest after surface staining. **b** Representative histogram plot for unstimulated (pink) vs NP + Mi + CpG (blue) for the upregulation of costimulatory molecules CD40, CD80, and CD86 on CD11c + bone marrow-derived dendritic cells from aged mice. **c** Estimates of log_2_ fold change (log_2_FC) and regulation tests for costimulatory molecules expressed on cell surfaces after stimulation by various adjuvants. All treatments were applied to cells from each of six mice (*n* = 6) with up to three technical replicates. The horizontal axis represents adjuvants; the vertical axis represents responses; color scale represents the log_2_FC; asterisks represent the degree of significance: **** q < 0.001, *** q < 0.01, ** q < 0.05. Dual adjuvant combinations are omitted from the main text for conciseness. Please refer to Supplementary Fig. S1 for results of dual adjuvants and controls

As an assessment of BMDC maturation through upregulation of costimulatory molecule expression, we investigated the stimulatory effect of several agonists on BMDCs generated from aged mice. BMDCs were gated based on FSC-A and SSC-A and doublets were excluded (Fig. 1a). Live (zombie NIR negative) cells were then selected, followed by selecting BMDCs that were positive for CD11c expression (Fig. 1a). As a representative example, histograms depicting the elevation of the cell surface markers CD40, CD80, and CD86 in NP + Mi + CpG-treated cells compared to untreated cells are shown in Fig. 1b. Immune stimulants, as single adjuvants and in combination with biomaterial carriers, differentially affected the expression of costimulatory molecules. To differentially evaluate these effects, we used a mixed linear model (described in Section 4.6), and in the results that follow, we denote adjusted *p*-values as q-values to indicate the degree of statistical significance. In comparison to unstimulated cells, treating the cells with CpG, LPS, MPLA, NP + Mi + CpG, NP + Mi + CDN, or NP + Mi + R848 increased the surface expression of all costimulatory molecules measured (i.e., CD40, CD80, and CD86; Fig. 1c; q < 0.001). In contrast, CDN upregulated only CD40 (q < 0.001), MDP upregulated CD40 (q < 0.001) and CD80 (q < 0.01), and FLA-BS upregulated CD40 (q < 0.05) and CD80 (q < 0.05). Moreover, cells treated with NP and Mi did not differ significantly from untreated controls in terms of their cell surface expression (Fig. 1c). “Dual” combinations of CDN and CpG with either NP or Mi were also included as treatment groups (see supplement, Fig. S1), but because these combinations did not outperform either single adjuvant or triple combinations, they are not discussed further in the main manuscript.

### Cytokine and chemokine secretion

In order to more clearly present the findings from a large panel, the cytokines were categorized as: (i) cytokines associated with T cell activation (IL-12p70, IFNα, IL-6, IFNγ, IFNβ); (ii) proinflammatory cytokines (IL-1β, IL-1α, IL-6, TNFα, IL-12p40); and (iii) cytokines with regulatory effects on DCs (IL-10). We assessed levels of cytokines and chemokines from supernatants of aged BMDCS stimulated for 24 h with various stimulants and dual/triple combinations as mentioned previously.

#### Cytokines associated with T cell activation

**Fig. 2:**
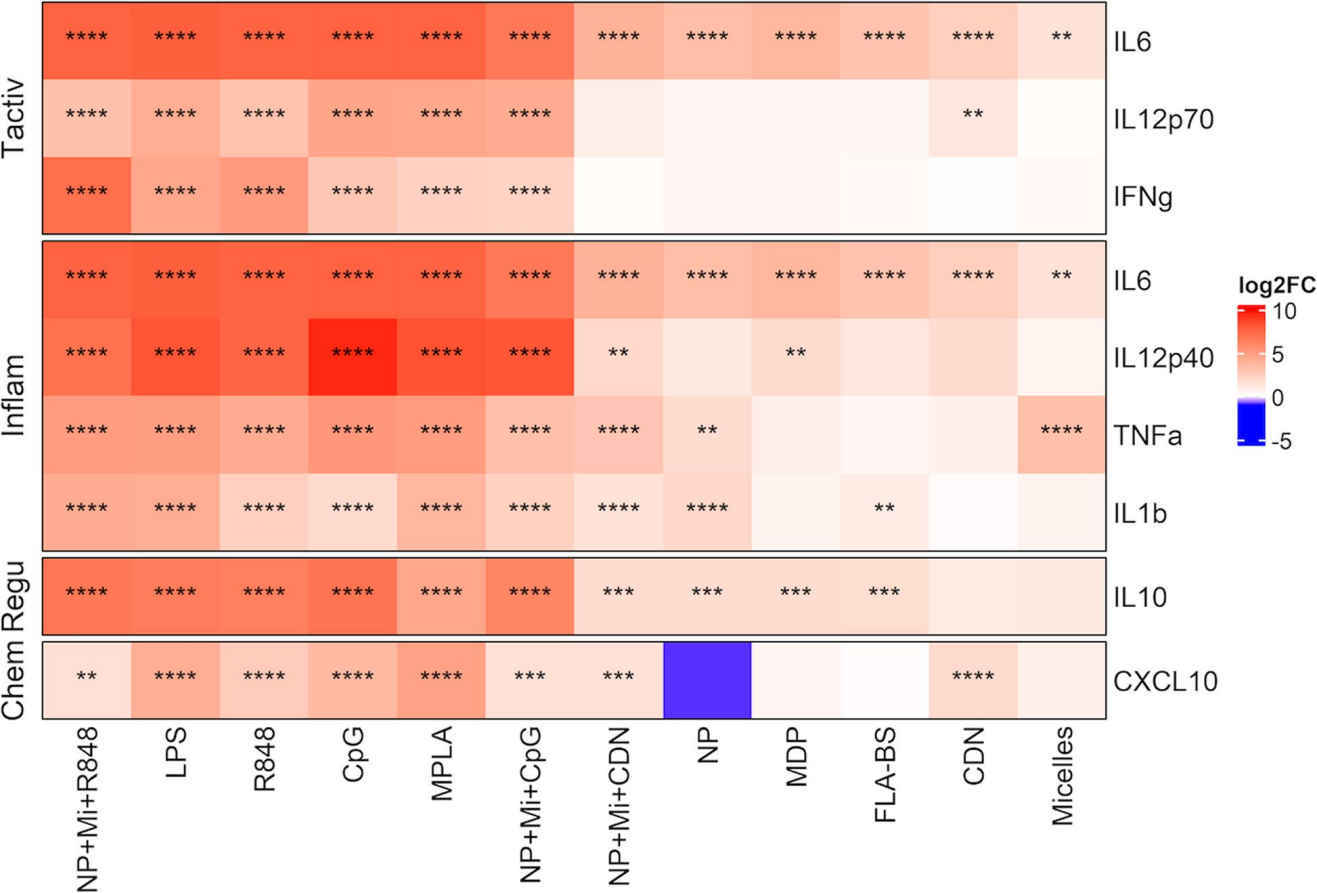
Estimates of log_2_ fold change (log_2_FC) and tests of regulation from mixed linear model analysis of cytokines produced by BMDCs generated from aged mice. Stimulants shown here include single adjuvants and their related triple combination. Each treatment was replicated with supernatants collected from cultures of cells from each of 3-6 animals. Results for dual adjuvants and their controls are shown in Supplementary Fig. S2a and chemokines are shown in Supplementary Fig. S2b

When compared to untreated cells, cells treated with NP + Mi + R848, LPS, R848, CpG, MPLA, and NP + Mi + CpG significantly increased IL-12p70 production (q < 0.001). CDN also increased IL-12p70 secretion (q < 0.05). We observed no significant impact of NP + Mi + CDN, NP, MDP, FLA-BS, or Mi on IL-12p70 production. NP + Mi + R848, LPS, R848, CpG, MPLA, and NP + Mi + CpG increased IFNγ (q < 0.001). However, NP + Mi + CDN, NP, MDP, FLA-BS, CDN, and Mi had no detectable effect on IFNγ. All stimulants generated considerable levels of IL-6 compared to untreated cells (q < 0.001 for all treatments except CDN q < 0.05) (Fig. 2). We also measured an expanded panel of T cell activating cytokines in a subset of cells that included IFNβ and IFNα (Sup. Figure 2b). IFNβ was strongly induced by LPS (q < 0.001), CpG (q < 0.001), MPLA (q < 0.001), NP + Mi + CpG (q < 0.001), NP + Mi + CDN (q < 0.001) and NP + Mi + R848 (q < 0.01). We observed no impact of NP, MDP, R848, and CDN on IFNβ production. However, FLA-BS and Mi appeared to downregulate IFNβ but there is no statistical evidence suggesting the downward regulation is significant. In addition, LPS, CpG, MPLA, NP + Mi + CpG, NP + Mi + CDN, and CDN strongly increased the production of IFNα (q < 0.001). R848 (q < 0.01) and NP + Mi + R848 (q < 0.05) also increased IFNα production relative to untreated cells. We observed no significant impact of NP, MDP, FLA-BS, and Mi on IFNα production.

#### Proinflammatory cytokines

NP + Mi + R848, LPS, R848, CpG, MPLA, NP + Mi + CpG, and NP + Mi + CDN and NP increased IL-1β production (q < 0.001). FLA-BS also upregulated IL-1β production, though not as a strongly (q < 0.05). MDP, CDN, and Mi had no effect on IL-1β production. TNFα was produced in large quantities by cells treated with NP + Mi + R848, LPS, R848, CpG, MPLA, NP + Mi + CpG, and NP + Mi + CDN and Mi (q < 0.001). TNFα production was likewise increased by NP treatment (q < 0.05). MDP, FLA-BS, and CDN had no effect on TNFα production compared to untreated cells. LPS, R848, CpG, MPLA, NP + Mi + CpG, and NP + Mi + R848 promoted the production of IL-12p40 (q < 0.001). NP + Mi + CDN and MDP both increased IL-12p40 secretion (q < 0.05). However, NP, FLA-Bs, CDN, and Mi had no effect on IL-12p40 production (Fig. 2). LPS, CpG, and R848 increased IL-1α production (q < 0.05), while NP + Mi + CDN inhibited IL-1α expression. MPLA, NP + Mi + R848, NP + Mi + CpG, NP, MDP, FLA-BS, Mi, and CDN did not stimulate IL-1α production (Fig. S2b).

#### Regulatory cytokines

NP + Mi + R848, LPS, R848, CpG, MPLA, NP + Mi + CpG, and NP + Mi + CDN increased IL-10 production (q < 0.001). NP + Mi + CDN, NP, MDP, and FLA-BS also increased IL-10 production (q < 0.01), though less strongly. There was no effect of Mi and CDN on IL-10 production (Fig. 2). Only Mi increased the production of CCL2 (q < 0.05) while CpG downregulated the production of CCL2 (Fig. S2b).

#### Chemokines

LPS, R848, CpG, MPLA, and CDN treatment enhanced CXCL10 levels compared to untreated cells (q < 0.001). CXCL10 production was also increased by NP + Mi + CpG, NP + Mi + CDN (q < 0.01), and NP + Mi + R848 (q < 0.05) stimulation. We did not observe any impact of Mi, MDP, or FLA-BS on the production of CXCL10. However, CXCL10 was downregulated by NP (Fig. 2). An expanded panel of chemokines was also assessed on a subset of cells (Sup. Figure 2b). Dual treatments (i.e., NP + CpG, Mi + CpG, NP + CDN and Mi + CDN) were also evaluated, but none of these combinations were superior to single adjuvant or triple combinations (Fig. S2a).

### Principal Component Analysis (PCA)

**Fig. 3:**
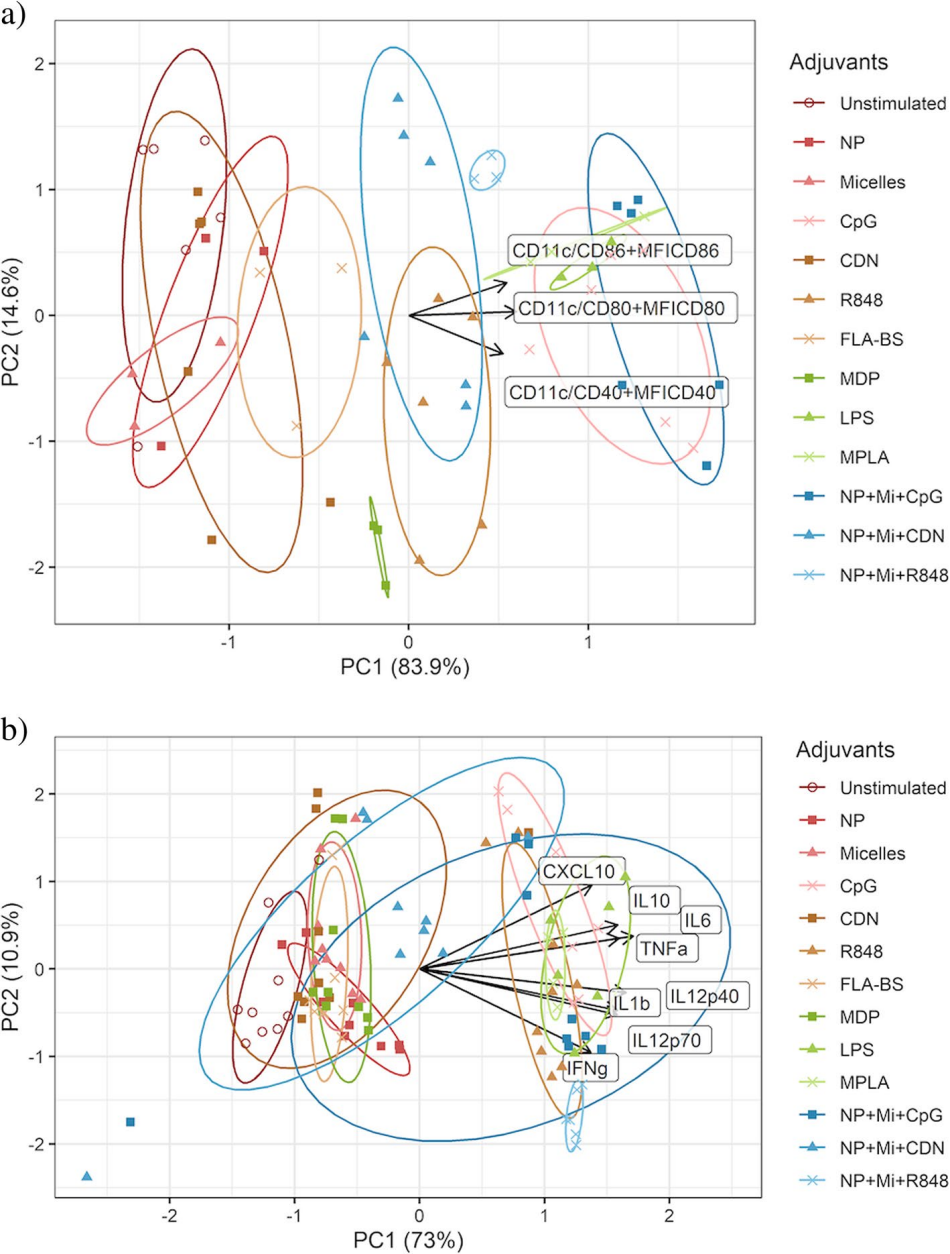
**a** PCA analysis of costimulatory molecule expression for the combined datasets of all experiments. Dual adjuvant groups are omitted in this analysis. The response names near arrows represents abbreviations as follows. The responses are *Cd11c + Cd86+, CD11c + CD80+,* and *CD11c + CD40+* from top to bottom. The lengths of the arrows in this figure were scaled down for readability. **b** PCA analysis of cytokines for the combined datasets of all experiments. Dual adjuvants are omitted in this analysis. The responses are CXCL10, IL10, IL6, TNFα, IL12p40, IL1b, IL12p70, IFNγ from top to bottom

To facilitate the clustering of the adjuvants’ effects compared to unstimulated cells, we performed PCA for costimulatory molecules and cytokines separately. All responses were log-transformed (log(Y + 1)) and standardized. The biplots in Fig. 3a and b show arrows (loading plot) that represent responses to treatment. The direction of the arrows shows how much weight each response has on the first two principal components (PCs). Each point on the PCA plot represents the response of BMDCs from a single aged mouse to a single adjuvant or adjuvant-biomaterial combination. The points are colored to represent different adjuvants and their combinations, and their location is determined by their first two PC scores, where similarities are present with respect to location for points corresponding to the same adjuvant. Finally, the biplot (loading plot + PCA plot) shows how one adjuvant affects responses compared to that of unstimulated cells by examining the projected distance and direction between the corresponding ellipse covering most points corresponding to the adjuvant and the ellipse covering most points for the unstimulated cells.

**Fig. 4:**
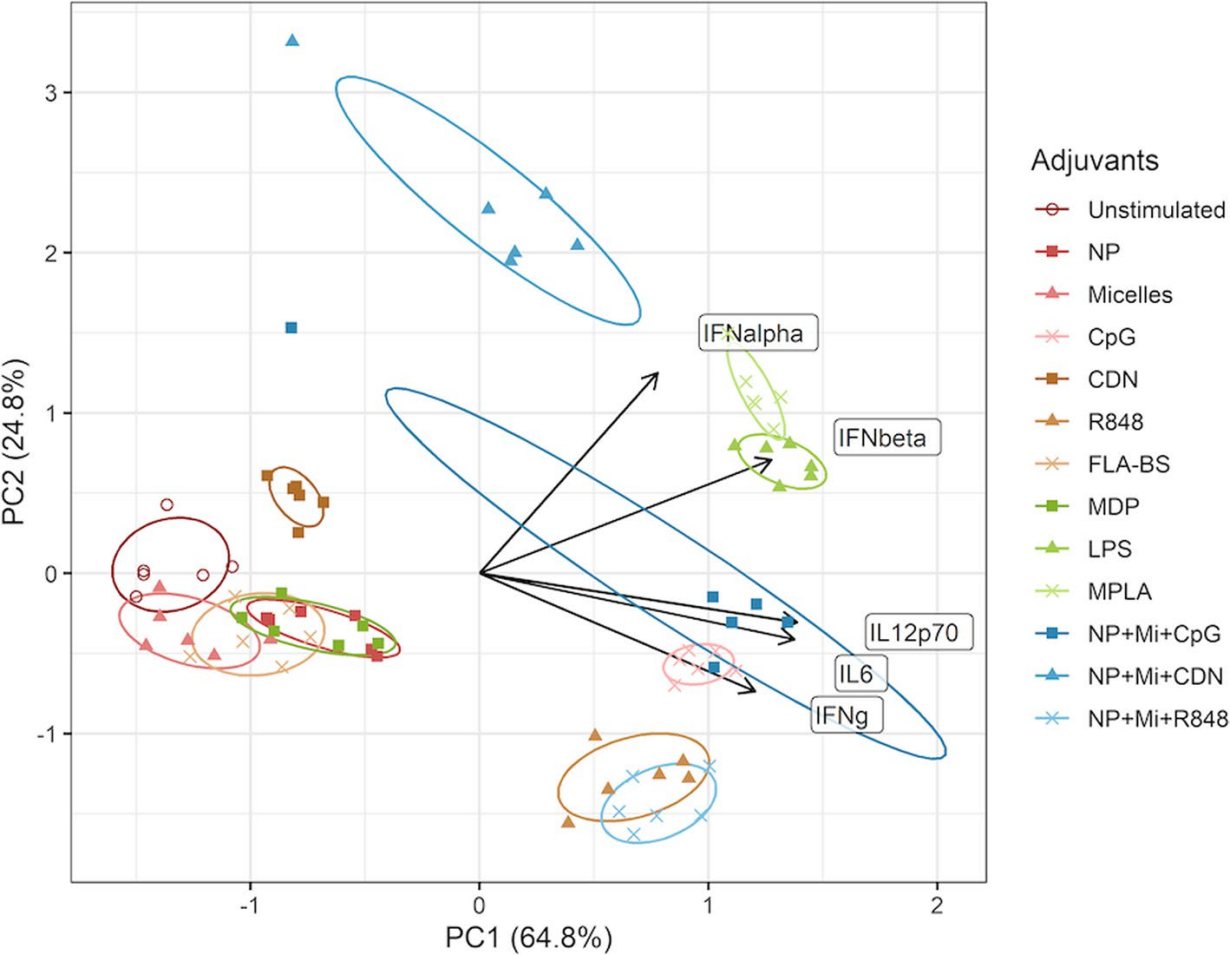
PCA for cytokines associated with T cell activation (CD4^+^ Tfh and CD8^+^ memory). The responses are IFNα, IFNβ, IL-6, IL-12p70, and IFNγ from top to bottom

**Fig. 5:**
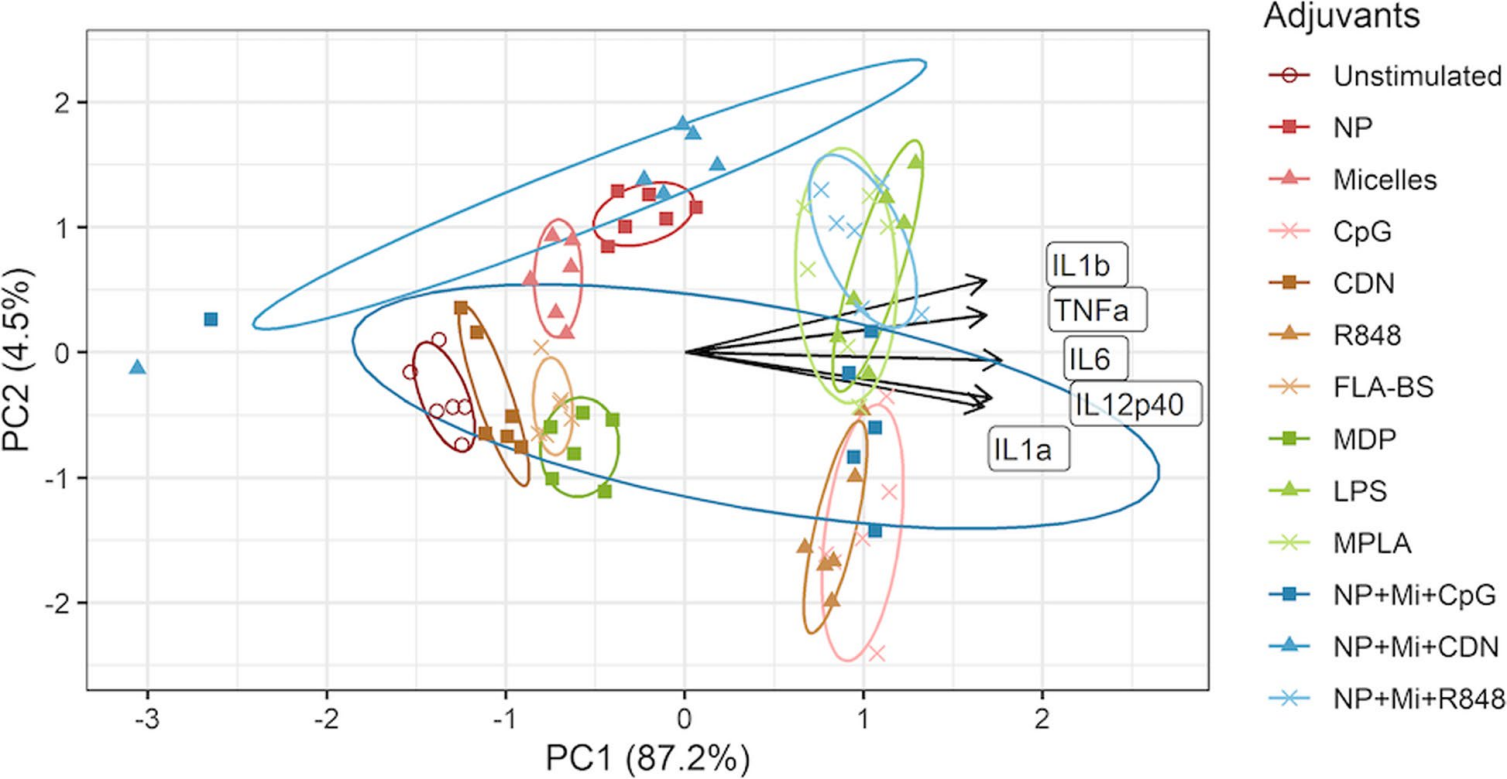
PCA for proinflammatory cytokine secretion. The responses are IL-1β, TNFα, IL-6, IL-12p40, and IL-1α from top to bottom

Figure 3a shows that the points representing cells stimulated by NP + Mi + CpG, CpG, LPS, MPLA, and NP + Mi + R848 are to the right of the unstimulated points, indicating that they contribute to the increase in responses in the direction of the first PC when compared to unstimulated cells. The horizontal projection of the arrows shows that the first PC is composed of a nearly equal-weighted sum of all three responses (i.e., CD80, CD86, and CD40 expression). Similarly, LPS, MPLA, CpG, R848 NP + Mi + R848 and NP + Mi + CDN are also observed to the right of the unstimulated cells in Fig. 3b. We calculated the differences in PC scores between the various adjuvants and the control. We observed a potential cluster of MPLA, LPS, CpG, and NP + Mi + CpG that increase costimulatory expression compared to other adjuvants (Table 2 and Fig. S3). In terms of cytokines, we identified a cluster of NP + Mi + CpG, R848, MPLA, CpG, LPS, and NP + R848 + Mi (Table 3 and Fig. S4). Finally, we performed a second PCA analysis for the cytokines categorized as either T cell activating (Fig. 4) or proinflammatory (Fig. 5). For the former, a cluster of NP + Mi + CpG, R848, NP + Mi + R848, CpG, MPLA, and LPS was identified that produced cytokines linked with T cell activation (Table 4 and Fig. S5). For the latter, a potential cluster of NP + Mi + CpG, MPLA, CpG, NP + Mi + R848, and LPS was identified, with R848 being the most proinflammatory (Table 5 and Fig. S6).

**Table 2:** Differences in PC scores and Euclidean distance (in the space defined by PC1 and PC2) between treatments and control (untreated cells) for costimulatory molecule expression. We observe a cluster of adjuvants (MPLA, LPS, CpG, and NP + Mi + CpG) that suggest greater upregulation of costimulatory molecule expression than the other adjuvants

Adjuvants	PC1(Adjuv)-PC1(Media)	PC2(Adjuv)-PC2(Media)	Distance(PC1,PC2)
NP	0.32	−0.45	0.55
CDN	0.42	−0.61	0.74
Micelles	−0.1	−0.82	0.82
FLA-BS	1.1	−0.51	1.21
NP + Mi + CDN	2.18	−0.15	2.19
MDP	1.81	−1.69	2.47
R848	2.32	−0.97	2.52
NP + Mi + R848	2.76	0.29	2.78
MPLA	3.54	−0.09	3.54
LPS	3.65	−0.19	3.66
CpG	3.95	−0.58	4.00
NP + Mi + CpG	4.25	−0.44	4.27

**Table 3:** PC score differences and Euclidean distance (in the space defined by PCs 1 and 2) between treatments and control (untreated cells) for cytokine production. We observe a cluster of adjuvants (NP + Mi + CpG, R848, MPLA, CpG, LPS, and NP + R848 + Mi) that may lead to greater cytokine production than other adjuvants

Adjuvants	PC1(Adjuv)-PC1(Media)	PC2(Adjuv)-PC2(Media)	Distance(PC1,PC2)
FLA-BS	1.15	0.14	1.16
Micelles	1.25	0.54	1.37
MDP	1.39	0.32	1.42
CDN	1.3	0.62	1.44
NP	1.53	−0.24	1.55
NP + Mi + CDN	2.16	0.68	2.27
NP + Mi + CpG	4.66	0	4.66
R848	5.27	0.07	5.27
MPLA	5.52	0.24	5.53
CpG	5.51	0.89	5.59
NP + Mi + R848	5.89	−1.37	6.05
LPS	6.11	0.34	6.12

**Table 4:** PCA for cytokines associated with T cell activation (CD4^+^ Tfh and CD8^+^ memory). Shown are PC score differences and Euclidean distance (in the space defined by PC1 and PC2) between treatments and control (untreated cells). We observed a cluster of adjuvants (NP + Mi + CpG, R848, NP + Mi + R848, CpG, MPLA, and LPS) that may have greater production of cytokines associated with T cell activation

Adjuvants	PC1(Adjuv)-PC1(Media)	PC2(Adjuv)-PC2(Media)	Distance(PC1,PC2)
Micelles	0.15	−0.46	0.48
FLA-BS	0.69	−0.48	0.84
CDN	0.99	0.47	1.09
MDP	1.12	−0.43	1.2
NP	1.2	−0.44	1.28
NP + Mi + CDN	2.5	2.53	3.55
NP + Mi + CpG	3.89	−0.06	3.89
R848	3.64	−1.48	3.93
NP + Mi + R848	3.84	−1.66	4.18
CpG	4.15	−0.69	4.21
MPLA	4.6	1.21	4.76
LPS	4.81	0.72	4.86

**Table 5:** PCA for proinflammatory cytokine secretion. Shown are PC score differences and Euclidean distance (in the space defined by PC1 and PC2) between treatments and control (untreated cells). We observed a cluster of adjuvants (NP + Mi + CpG, R848, MPLA, CpG, NP + Mi + R848, and LPS) that appear to be inducing the most proinflammatory cytokines

Adjuvants	PC1(Adjuv)-PC1(Media)	PC2(Adjuv)-PC2(Media)	Distance(PC1,PC2)
CDN	0.58	0	0.58
FLA-BS	1.27	−0.04	1.27
Micelles	1.33	0.45	1.4
MDP	1.58	−0.19	1.59
NP + Mi + CDN	1.7	0.76	1.86
NP	2.33	0.69	2.43
NP + Mi + CpG	3.64	−0.04	3.64
R848	4.56	−0.49	4.59
MPLA	4.69	0.46	4.71
CpG	4.88	−0.51	4.91
NP + Mi + R848	4.89	0.59	4.92
LPS	5.06	0.49	5.08

## Discussion

Aging is associated with impaired immunological function and a higher risk of infection [83]. Although vaccination is a tried-and-true method of preventing infections in older adults, it has been shown that the initial antibody response to vaccination declines with age and that older persons have a shorter immunization duration [84–86], and that pre-existing inflammation leads to poor antigen presentation [15]. The poor responsiveness of the aging immune system may be addressed by increasing both the innate and adaptive immunological responses to vaccination and by counteracting the low-grade inflammatory state that could otherwise impede vaccine responses in older persons [40].

DCs are potential targets to improve immunity because of their shorter life spans and their location upstream of the activation of T and B lymphocytes [18, 26, 87]. Recent years have seen growing evidence supporting the role of DC function in immunological disorders, and promising research into targeting DCs for treating several diseases [88]. Adjuvants have an important role in increasing the humoral and cellular immune responses elicited by vaccines by triggering local proinflammatory cytokine production, activating innate immune cells, and stimulating antigen presentation [89]. However, when administered by themselves, many of them display adverse effects such as overt inflammation [90]. Our studies show that optimal stimulation of aged BMDCs may be achieved by combining adjuvants with our polymeric adjuvant nano-carriers without exacerbating inflammation. Through a multi-faceted approach of assessing costimulatory expression and cytokine production, we determined which combination nanovaccines were most effective at stimulating APC generated from aged mice and minimizing induction of pro-inflammatory cytokine responses.

Our results demonstrate that various TLR agonists (TLR2, TLR9, TLR7-8) upregulate CD40, CD80, and CD86 expression on the surface of aged BMDCs (Fig. 1). This observation is consistent with previous work showing that aging does not impair BMDCs’ capacity to generate an immunological response to TLR activation [91]. However, we found that FLA-BS (TLR 5 agonist) upregulated CD40 and CD80 but not CD86. Similarly, MDP, a ligand for the intracellular NOD2 receptor, upregulated both CD40 and CD80 but not CD86. Both flagellin and MDP have been shown to upregulate CD86 in human monocyte-derived DCs [92]; however, these studies differed in either/both the cell type utilized or using cells from young adults. We have previously shown that NP activate BMDCs from young mice by increasing CD40, CD80, and CD86 expression [93]; however, we did not observe this effect in the current study with aged mice, though this may be, in part, due to differences in experimental design. In prior studies the cells were incubated for 48 h with NP, suggesting that NP may activate DC at a later time point. CDN, a stimulator of interferon genes (STING), only increased CD40 expression, but when combined with NP and Mi, showed a synergistic impact resulting in a substantial rise in CD80 and CD86 expression. Similarly, NP and Mi in combination with CpG (TLR 9) and R848 (TLR 7-8) significantly increased all cell surface markers.

The capacity of stimulants to increase aged BMDC immune effector functions was assessed by measuring cytokines linked with T cell activation and inflammation. The intensity and the direction of the immune response are determined by the processes through which certain stimulants exert their adjuvanticity. We found that LPS, MPLA, CpG, and R848 all generated high levels cytokines associated with T cell activation while simultaneously increasing levels of proinflammatory cytokines (Fig. 2). These findings align with previous work that suggests that TLR signals induce proinflammatory cytokines such as IL-1ß, TNFα and IL-12p40 [94]. FLA-BS and MDP had a modest effect on the production of cytokines but since they did not elicit the greatest response, they are not discussed in detail. Our results demonstrated that CDN-induced responses produced less inflammatory cytokines than the other TLR agonists, which is consistent with literature showing that CDN does not have the same inflammatory profile as other TLR agonists [95]. IFN-α and IFN-β assist in expanding antiviral pathways and play an important role in building adaptive immunity to viral infection by increasing T cell activation and survival [96]. Additionally, IFN-α and IFN-β have a crucial function in promoting DC responses [97, 98]. We assessed IFNα/β (Sup. Figure 2b) and found that CpG, LPS, and MPLA all substantially upregulated IFNα/β production. These findings are consistent with previous research showing that treatment with a TLR agonist results in an increase in the synthesis of IFN-α and IFN-β in mouse and human cells [99, 100]. On the other hand, both CDN and R848 increased IFNα but not IFNβ. This result is consistent with earlier research, with one study reporting that TLR7/8 signaling increases IFNα [100]. However, when R848 and CDN were combined with Np and Mi, IFN-β production surged (Sup. Figure 2b). Similar results were obtained when combining NP and Mi with either CpG, CDN, or R848, on the secretion of various cytokines. While NP and Mi were not immunostimulatory on their own, these data show that the biomaterials used in this study positively contribute to the generation of these vital cytokines (Signal 3) when combined with the appropriate adjuvants.

Through mixed linear model analysis, we obtained new semi-quantitative insights into to how impactful the various adjuvant treatments were with respect to DC activation. However, we wanted to further understand the interplay between different adjuvants and combinations thereof with respect to their ability to stimulate costimulatory molecule expression and cytokine production. Therefore, we performed PCA and calculated PC scores between the various treatments and the control. These analyses allowed us to simultaneously analyze cytokines and costimulatory molecules in an effort to identify the “just right” combinations for adjuvants and nanoscale carriers (Figs. 3, 4 and 5 and Tables 2, 3, 4 and 5). These data show that while CpG has a propensity to substantially induce secretion of cytokines associated with T cell activation, it also induces higher proinflammatory cytokine production (Tables 4 and 5). On the other hand, cytokines associated with T cell activation were also enhanced by combining NP and Mi with CpG, without leading to excessive inflammation. This suggests that the inflammatory effects of CpG may be mitigated by formulating it with NP and Mi. Indeed, we have previously shown that the combination of CpG and NP offers robust and “universal” protection against influenza A virus when administered intranasally in young mice [47]. We also showed that while CDN is not highly inflammatory on its own, in combination with NP and Mi, the combination takes on a more proinflammatory profile and is more likely to be associated with cytokines that activate T cells (Tables 4 and 5). This observation is supported by our previous studies in which we showed that formulating CDN with nanovaccines protected both young and aged mice against influenza A infection [49].

The current studies offer a foundation to support the concept of judiciously combining adjuvants with nanoscale delivery platforms to induce a “just right” immune response in aged immune systems. We have shown that combining our nanoscale carriers with certain TLR and/or STING agonists can positively impact BMDCs generated from aged mice. We suggest that nanoparticles and micelles are working together in conjunction with these adjuvants to reduce the production of inflammatory cytokines while simultaneously boosting the production of T cell-related cytokines. Vaccines need to be developed that appropriately stimulate the aged immune system by balancing the induction of robust antibody and T cell responses with appropriately low levels of inflammation so as to not diminish the overall immune response to vaccination. This balanced approach may be particularly important for mucosal vaccines delivered to sites of local inflammaging, such as the lung. In this context, combination approaches that formulate small molecule adjuvants with nanoparticles and micelles may provide a promising pathway forward to design next generation vaccines for older adults. While these results provide new insights into rational adjuvant selection and suggest that the combination of specific adjuvants and biomaterials provides enhanced immune activation while minimizing inflammation, caution should be used when inferring how these treatments may affect the immune system in vivo*.* We note that the in vitro experimental design used herein is a limitation to understanding responses at the level of the whole organism, but these studies provide a means for identifying combinations that show the most promise for future in vivo work.

## Materials and methods

### Study system

The effects of combinations of polymeric nanoparticles, micelles, and various small molecule agonists on the activation of bone marrow derived DCs (BMDCs) harvested from the femur and tibia of aged (≥20 months) C57BL/6 male mice (*n* = 6) were studied. Bone marrow cells were cultured as outlined in Lutz et al. [101]. Briefly, cells were cultured in 10 mL RPMI 1640 medium supplemented with 100 U/mL penicillin, 100 mg/mL streptomycin, 2 mM glutamine, 10% FBS, and 20 ng/mL granulocyte-macrophage colony-stimulating factor (GM-CSF, Cat. #FB0875711Z, Peprotech, Rocky Hill, NJ) at approximately 5 × 10^6^ cells per 100 mm plate. On day 3 of culture, 10 mL of medium containing GM-CSF was added. On days 6 and 8 of the culture period, approximately half of the total volume of medium was removed and replaced with freshly supplemented RPMI. On day 10 plates were gently rinsed to harvest non-adherent DCs for assessment of activation and costimulatory expression. Animals were obtained from Jackson Laboratory (Bar Harbor, ME) and maintained at Iowa State University following IACUC protocol #IACUC-20-199.

### Stimulants

#### Polyanhydride NP synthesis

Polyanhydride monomers were synthesized using CPTEG and CPH as described previously [44, 45]. Using these monomers, a 20:80 CPTEG:CPH copolymer was synthesized by melt polycondensation as previously reported [45]. Using ^1^H nuclear magnetic resonance spectroscopy (^1^H NMR; DXR 500 Bruker, Billerica, MA) the final copolymer composition (23:77), molecular weight (11,000 g/mol), and purity were determined. Nanoparticles were synthesized using a solid-oil-oil double emulsion technique as previously described [102]. In brief, a 20 mg mL^− 1^ solution of 20:80 CPTEG:CPH copolymer dissolved in methylene chloride was sonicated for 30 s to ensure complete dissolution of the polymer. The solution was then poured into chilled pentane (− 10 °C; 1:250 methylene chloride:pentane) and the resulting particles collected via vacuum filtration. Nanoparticle morphology and size (∼200 nm) were verified with scanning electron microscopy (FEI Quanta 250, FEI, Hillsboro, OR).

#### PBC mi synthesis

The pentablock copolymer (PDEAEM–PEO-PPO–PEO-PDEAEM) was synthesized following our previously published protocol [46]. In short, atom transfer radical polymerization (ATRP) was used to synthesize the pentablock copolymer using a brominated Pluronic®-F127 as macroinitiator. The Pluronic F127 was dissolved in tetrahydrofuran and reacted overnight with triethylamine and 2-bromoisobutyryl. To validate the end group functionalization, the product was precipitated in n-hexane and analyzed by ^1^H NMR. The macroinitiator and DEAEM monomer were then reacted by ATRP to synthesize the pentablock copolymer, with copper(I) oxide nanoparticles acting as the catalyst and N-propylpyrilidine methanamine acting as the complexing ligand [103]. The pentablock copolymer was characterized using ^1^H NMR to determine purity and molecular weight (15,000 g/mol). The pentablock copolymer was dissolved in aqueous solution (12.5 μg/mL) to yield micelles.

#### Adjuvants

Lipopolysaccharide (LPS) (Cat. # L6529-1MG, Sigma Aldrich), Class B CpG oligonucleotide (CpG) (Cat.# tlrl-1668 InvivoGen), bis-(3′-5′)-cyclic dimeric guanosine monophosphate (c-di-GMP) (CDN) (Cat.# tlrl-nacdg, InvivoGen), muramyl dipeptide (MDP) (Cat.# tlrL-mdp InvivoGen), ultrapure flagellin from *B. subtilis* (FLSA-BS) (Cat.# tlrl-pbsfla InvivoGen), monophosphoryl lipid A (MPLA) (Cat.# *L6895 Sigma*-Aldrich) and R848 (Cat.# tlrl-r848 InvivoGen) were purchased and used.

### In vitro APC stimulation

BMDCs were seeded at 2.6 × 10^5^ cells per well in supplemented RPMI (as previously described) into 96-well round bottom tissue culture-treated microtiter plates (Cat. # FB0875711Z, Fisherbrand) at a volume of 200 μL per well. BMDCs were stimulated for 24 h with the adjuvants and concentrations shown in Table 1 as well as with double and triple combinations of adjuvants and biomaterials (e.g., NP + CpG, Mi + R848, NP + Mi + CpG, NP + Mi + CDN, NP + Mi + R848). Following stimulation, supernatants were collected and stored at − 20 °C until use for quantification of cytokines. Cells were collected and assessed for surface marker expression.

### Costimulatory molecule expression

Costimulatory molecule expression was assessed via flow cytometry. BMDCs were generated from the bone marrow of six aged mice across four separate days. Following culture, BMDCs were incubated with each treatment (i.e., single adjuvants, double, and triple combinations) for 24 h before assessment. Briefly, BMDCs (2.6 × 10^5^ per well in a 96-well plate) were stained with Zombie NIR dye (Cat. #423105, BioLegend), washed, then blocked with FcR blocking reagent (1 μL/well; 130-092-575 Miltenyi Biotech). Cells were then stained with anti-CD11c (BD Biosciences 557,401), anti-CD40 (BD Biosciences, 553,723), anti-CD80 (BD Biosciences, 560,526), and anti-CD86 antibodies (BD Biosciences, 560,581) by adding 0.25 μL/well of each monoclonal antibody. Following labeling, DCs were transferred to polystyrene tubes and fixed using BD stabilizing fixative (BD Bioscience, Franklin Lakes, NJ). Data were collected on a FACSCanto II flow cytometer (BD Bioscience, Franklin Lakes, NJ) and analyzed using FlowJo (Flowjo™10 LLC).

### Cytokines

A total of 23 cytokines/chemokines were quantified using a multiplexed, bead-based immunoassay (Milliplex mouse cytokine/chemokine magnetic bead panel, Cat. # MCYTOMAG-70 K, MTH17MAG-47 K, and MECY2MAG-73 K; Millipore, St Charles, MO) on a Luminex detection platform (Luminex, Austin, TX). All treatments (Table 1) were applied to cells from each of six mice and supernatants were collected from culture (*n* = 6 mice) following a 24 h incubation period. As cells were cultured on four separate days, supernatants were collected and stored at − 20 °C until use. Two panels of cytokines/chemokines were run using 25 μL supernatant per well, undiluted. For each plate, there were three biological replicates for each treatment. The second plate included an expanded panel of cytokines (see supplement Fig. S2b) and thus biological replicates for each cytokine/chemokine assayed ranged from *n* = 3 to 6. Assays were completed according to manufacturer protocol, with an overnight incubation (with agitation) at 4 °C.

### Statistical analyses

We performed a separate mixed linear model analysis for each dataset associated with costimulatory molecule expression and cytokine secretion. We also performed a mixed linear model analysis of the combined costimulatory molecule expression and cytokine secretion data. We present the results of these combined datasets in the manuscript and refer the reader to supplementary materials for the analysis of separate datasets. For each separate costimulatory molecule expression and cytokine secretion dataset, we modelled the log of each measured response plus one (i.e., log(Y + 1)) as a function of adjuvant fixed effects, normally distributed mouse random effects, and normal random errors. For each of the combined analyses, we added fixed effects for plates to the mixed linear model. As part of each mixed linear model analysis, we performed individual tests on the regulation of each log-transformed response for each adjuvant/combination compared to unstimulated cells. Specifically, we tested the difference between the linear coefficients of each adjuvant and the unstimulated cells. Furthermore, we use adjusted *p*-values, referred to as q-values, to control false discovery rate control (FDR) for the collection of tests within each dataset [104]. The procedure was implemented in R using the functions “lme4::lmer”,“lmerTest::contest” [105], “stats::p.adjust”, and “ComplexHeatmap::Heatmap” [106].

Additionally, we performed principal component analysis (PCA) to gain insights into clustering effects of single adjuvants and treatment combinations on the regulation of both costimulatory expression and cytokine production. Responses to treatment were log-transformed (log(Y + 1)) and standardized. As a means of facilitating the clustering, all treatment groups were compared to untreated cells. The numbers of clusters were selected by the silhouette method [107], followed by the identification of clusters with the k-means clustering method [108]. The procedure was implemented in R using the functions “stats::prcomp”, “factoextra::fviz_nbclust”, “stats::kmeans”, and “factoextra:: fviz_cluster” [109].

## Supplementary Information

**Additional file 1: Fig. S1.** Estimates of log_2_ fold change (log_2_FC) and regulation tests for costimulatory molecules expressed on CD11c^+^ cell surfaces after stimulation by single adjuvant, dual treatments, and controls. Color scale represents the estimated log_2_ fold change (log_2_ FC) in molecule expression to a specific treatment relative to untreated cells. Asterisks represent the degree of significance for a given treatment relative to untreated cells: **** q < 0.001, ** q < 0.05. Responses to treatments were measured for each of six mice (*n* = 6) with multiple replicates. **Fig. S2.** a. Estimates of log_2_ fold change (log_2_FC) and tests of regulation from mixed linear model analysis of cytokines. This panel includes single adjuvants and their related double, and triple combinations. The color scale represents the estimated log_2_ fold change (log_2_FC) of a given cytokine to a specific treatment relative to untreated cells. Asterisks represent the degree of significance for each treatment relative to untreated cells: **** q < 0.001, *** q < 0.01, ** q < 0.05. Each treatment was tested on cells harvested from each of *n* = 3-6 animals. b. Estimates of log_2_ fold change (log_2_FC) and tests of regulation from mixed linear model analysis of an expanded panel of cytokines. The color scale represents the estimated log_2_ fold change (log_2_FC) of a given cytokine to a specific treatment relative to untreated cells. Asterisks represent the degree of significance for each treatment relative to untreated cells: **** q < 0.001, *** q < 0.01, ** q < 0.05. Each treatment was tested on cells harvested from each of n = 3 animals on a single kit. **Fig. S3.** Pictorial representation of the data in Table 2. We observed a cluster of treatments (cluster 2, comprised of MPLA, LPS, CpG, and NP + Mi + CpG) that suggest a greater upregulation in costimulatory molecule expression than the other adjuvants. **Fig. S4.** Pictorial representation of the data in Table 3. We observed a cluster of treatments (cluster 1, comprised of NP + Mi + CpG, R848, MPLA, CpG, LPS, and NP + R848 + Mi) that suggests a stronger upregulation of cytokine secretion than the other adjuvants shown in cluster 2. **Fig. S5.** Pictorial representation of the data in Table 4. Cluster 1 (NP + Mi + CpG, R848, NP + Mi + R848, CpG, MPLA, and LPS) contains treatments which are most strongly associated with T cell activation. **Fig. S6.** Pictorial representation of the data in Table 5. Cluster 2 (NP + Mi + CpG, MPLA, CpG, NP + Mi + R848, LPS, and R848) contains treatments most strongly associated with proinflammatory cytokine secretion.

## Data Availability

The datasets used and/or analyzed during the current study are available from the corresponding author on reasonable request.
