# Supplementary material for: “Just right” combinations of adjuvants with nanoscale carriers activate aged dendritic cells without overt inflammation
Source: Immun Ageing. 2023 Mar 9;20:10. doi: 10.1186/s12979-023-00332-0 (PMC9996592; doi:10.1186/s12979-023-00332-0)
Supplement: Supplementary file 1 — Additional file 1: Fig. S1. Estimates of log2 fold change (log2FC) and regulation tests for costimulatory molecules expressed on CD11c+ cell surfaces after stimulation by single adjuvant, dual treatments, and controls. Color scale represents the estimated log2 fold change (log2 FC) in molecule expression to a specific treatment relative to untreated cells. Asterisks represent the degree of significance for a given treatment relative to untreated cells: **** q < 0.001, ** q < 0.05. Responses to treatments were measured for each of six mice (n = 6) with multiple replicates. Fig. S2. a. Estimates of log2 fold change (log2FC) and tests of regulation from mixed linear model analysis of cytokines. This panel includes single adjuvants and their related double, and triple combinations. The color scale represents the estimated log2 fold change (log2FC) of a given cytokine to a specific treatment relative to untreated cells. Asterisks represent the degree of significance for each treatment relative to untreated cells: **** q < 0.001, *** q < 0.01, ** q < 0.05. Each treatment was tested on cells harvested from each of n = 3-6 animals. b. Estimates of log2 fold change (log2FC) and tests of regulation from mixed linear model analysis of an expanded panel of cytokines. The color scale represents the estimated log2 fold change (log2FC) of a given cytokine to a specific treatment relative to untreated cells. Asterisks represent the degree of significance for each treatment relative to untreated cells: **** q < 0.001, *** q < 0.01, ** q < 0.05. Each treatment was tested on cells harvested from each of n = 3 animals on a single kit. Fig. S3. Pictorial representation of the data in Table 2. We observed a cluster of treatments (cluster 2, comprised of MPLA, LPS, CpG, and NP + Mi + CpG) that suggest a greater upregulation in costimulatory molecule expression than the other adjuvants. Fig. S4. Pictorial representation of the data in Table 3. We observed a cluster of treatments (cluster 1 [file 12979_2023_332_MOESM1_ESM.docx]

SUPPLEMENTARY INFORMATION

Ananya et al. “Just right” combinations of adjuvants with nanoscale carriers activate aged dendritic cells without overt inflammation


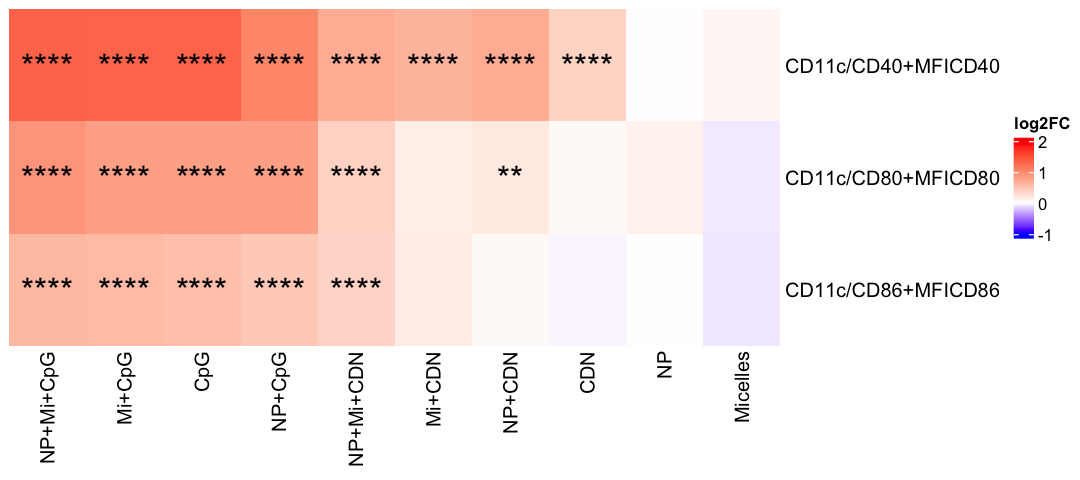


**Figure S1**. Estimates of log_2_ fold change (log_2_FC) and regulation tests for costimulatory molecules expressed on CD11c^+^ cell surfaces after stimulation by single adjuvant, dual treatments, and controls. Color scale represents the estimated log_2_ fold change (log_2_ FC) in molecule expression to a specific treatment relative to untreated cells. Asterisks represent the degree of significance for a given treatment relative to untreated cells: **** q<0.001, ** q<0.05. Responses to treatments were measured for each of six mice (n=6) with multiple replicates.


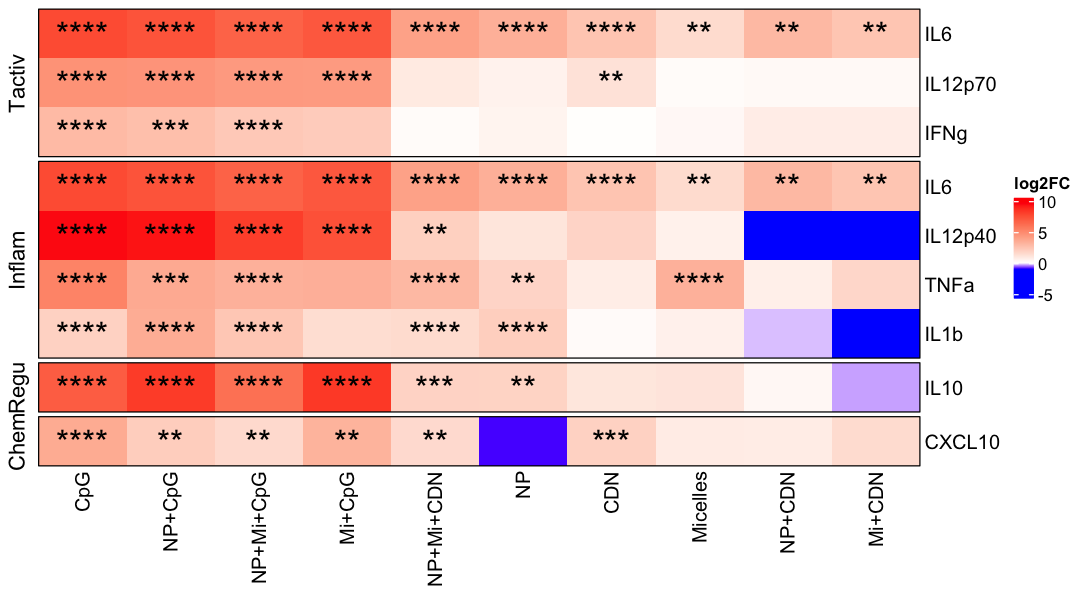


**Figure S2a.** Estimates of log_2_ fold change (log_2_FC) and tests of regulation from mixed linear model analysis of cytokines. This panel includes single adjuvants and their related double, and triple combinations. The color scale represents the estimated log_2_ fold change (log_2_FC) of a given cytokine to a specific treatment relative to untreated cells. Asterisks represent the degree of significance for each treatment relative to untreated cells: **** q<0.001, *** q<0.01, ** q<0.05. Each treatment was tested on cells harvested from each of n=3-6 animals.


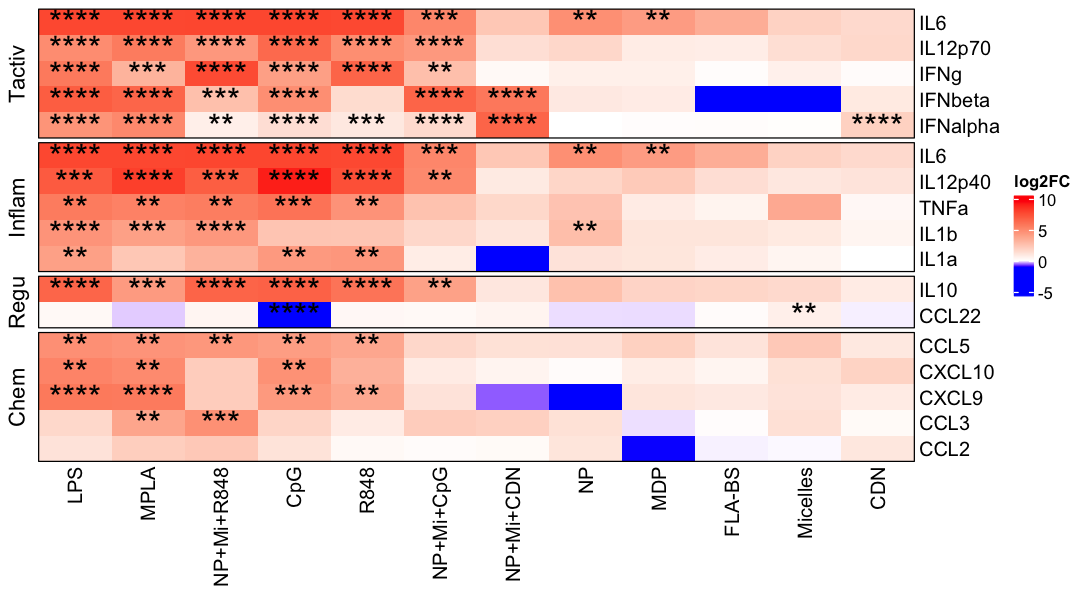


**Figure S2b.** Estimates of log_2_ fold change (log_2_FC) and tests of regulation from mixed linear model analysis of an expanded panel of cytokines. The color scale represents the estimated log_2_ fold change (log_2_FC) of a given cytokine to a specific treatment relative to untreated cells. Asterisks represents the degree of significance for each treatment relative to untreated cells: **** q<0.001, *** q<0.01, ** q<0.05. Each treatment was tested on cells harvested from each of n=3 animals on a single kit.


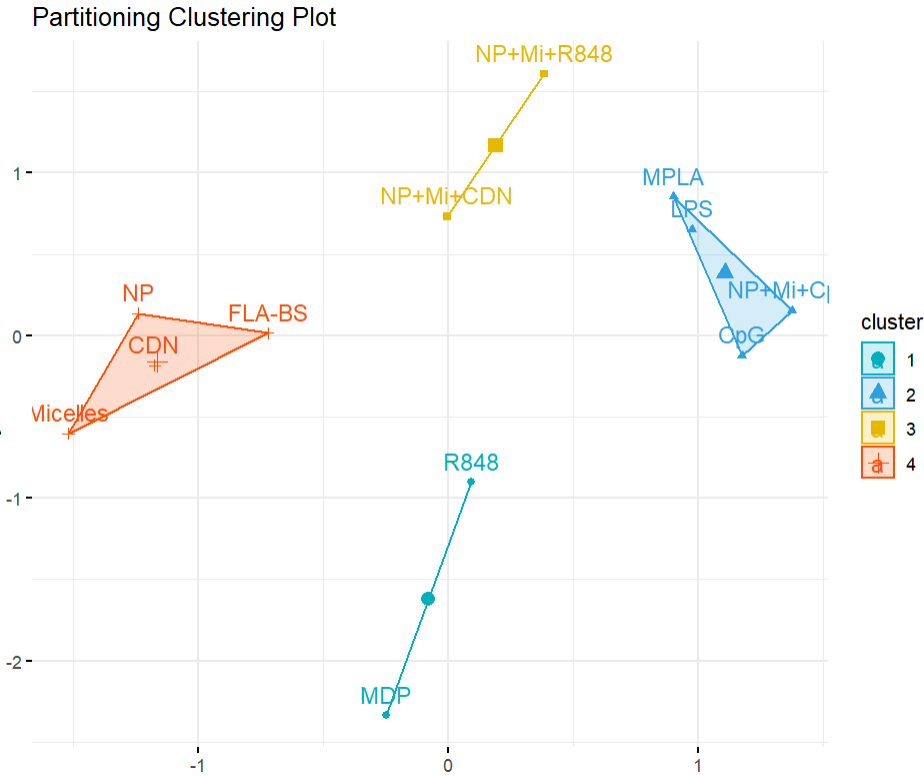


**Figure S3.** Pictorial representation of the data in Table 2. We observed a cluster of treatments (cluster 2, comprised of MPLA, LPS, CpG, and NP+Mi+CpG) that suggest a greater upregulation in costimulatory molecule expression than the other adjuvants.


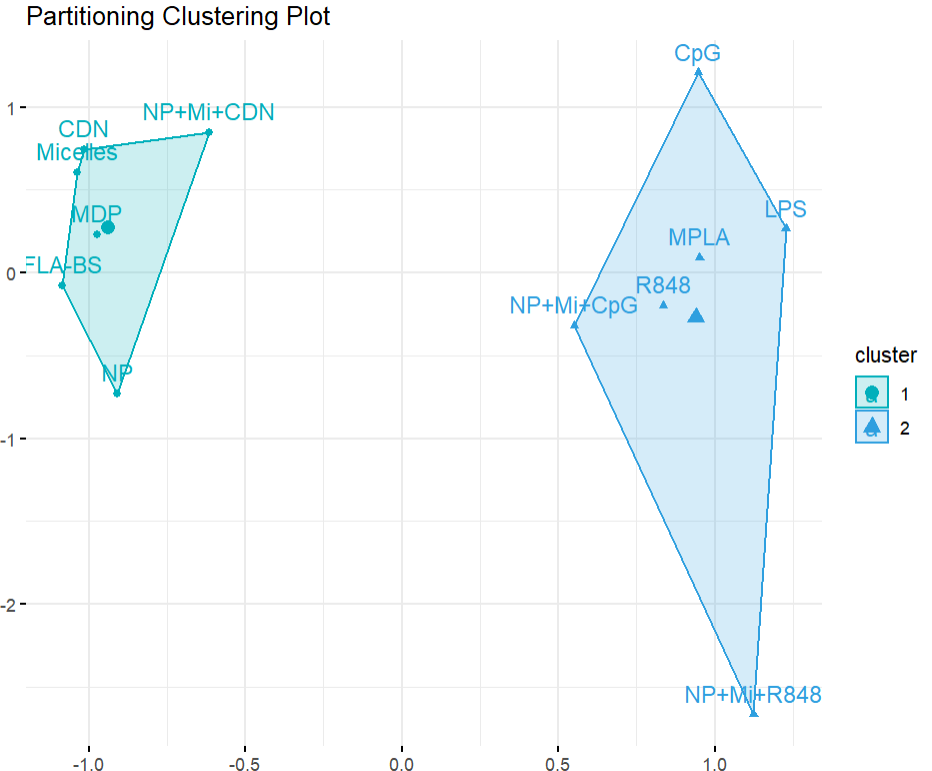


**Figure S4.** Pictorial representation of the data in Table 3. We observed a cluster of treatments (cluster 1, comprised of NP+Mi+CpG, R848, MPLA, CpG, LPS, and NP+R848+Mi) that suggests a stronger upregulation of cytokine secretion than the other adjuvants shown in cluster 2.


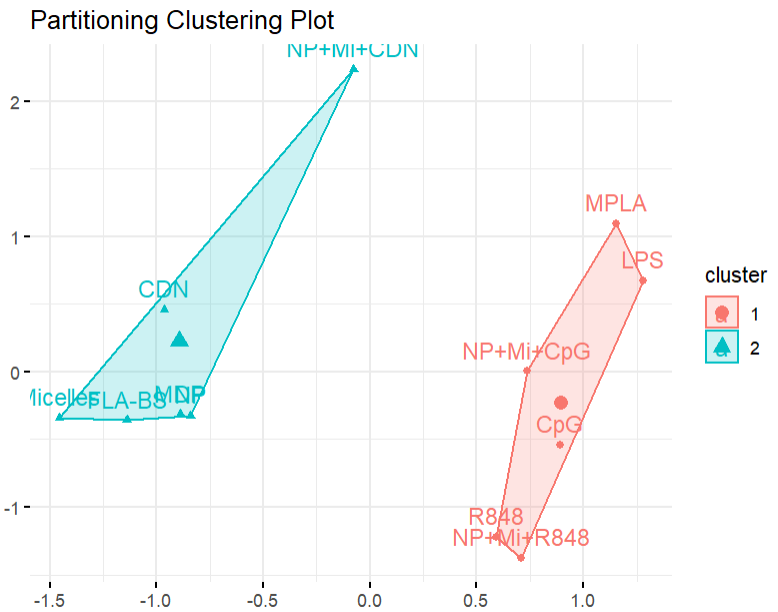


**Figure S5.** Pictorial representation of the data in Table 4. Cluster 1 (NP+Mi+CpG, R848, NP+Mi+R848, CpG, MPLA, and LPS) contains treatments which are most strongly associated with T cell activation.

*
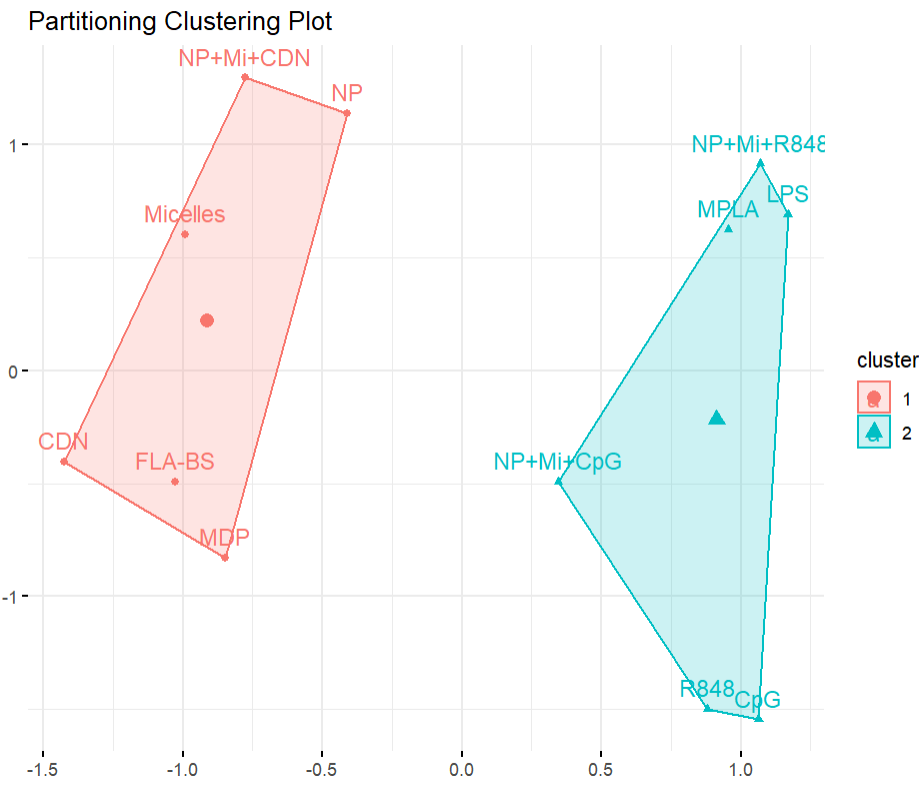
*

**Figure S6**. Pictorial representation of the data in Table 5. Cluster 2 (NP+Mi+CpG, MPLA, CpG, NP+Mi+R848, LPS, and R848) contains treatments most strongly associated with proinflammatory cytokine secretion.
